# Evaluation of a course to prepare international students for the United States Medical Licensing Examination step 2 clinical skills exam

**DOI:** 10.3352/jeehp.2017.14.25

**Published:** 2017-10-24

**Authors:** Rachel B. Levine, Andrew P. Levy, Robert Lubin, Sarah Halevi, Rebeca Rios, Danelle Cayea

**Affiliations:** 1 Department of Medicine, Johns Hopkins University School of Medicine, Baltimore, MD, USA; 2 Technion Faculty of Medicine, Technion Israel Institute of Technology, Haifa, Israel; Hallym University, Korea

**Keywords:** Curriculum, Educational measurement, Foreign medical graduates, Clinical competence

## Abstract

**Purpose:**

United States (US) and Canadian citizens attending medical school abroad often desire to return to the US for residency, and therefore must pass US licensing exams. We describe a 2-day United States Medical Licensing Examination (USMLE) step 2 clinical skills (CS) preparation course for students in the Technion American Medical School program (Haifa, Israel) between 2012 and 2016.

**Methods:**

Students completed pre- and post-course questionnaires. The paired t-test was used to measure students’ perceptions of knowledge, preparation, confidence, and competence in CS pre- and post-course. To test for differences by gender or country of birth, analysis of variance was used. We compared USMLE step 2 CS pass rates between the 5 years prior to the course and the 5 years during which the course was offered.

**Results:**

Ninety students took the course between 2012 and 2016. Course evaluations began in 2013. Seventy-three students agreed to participate in the evaluation, and 64 completed the pre- and post-course surveys. Of the 64 students, 58% were US-born and 53% were male. Students reported statistically significant improvements in confidence and competence in all areas. No differences were found by gender or country of origin. The average pass rate for the 5 years prior to the course was 82%, and the average pass rate for the 5 years of the course was 89%.

**Conclusion:**

A CS course delivered at an international medical school may help to close the gap between the pass rates of US and international medical graduates on a high-stakes licensing exam. More experience is needed to determine if this model is replicable.

## Introduction

International medical graduates (IMGs) make up approximately 25% of current trainees in United States (US) residency programs. A substantial subset (about 38%) of IMGs are US and Canadian citizens (US IMGs) who attend medical school abroad [1]. Most of these students desire to return to the US or Canada to complete residency training and practice medicine. US IMGs represent about 14% of all US residency applicants [1]. To enter graduate training in the US, IMGs must be certified by the Educational Commission for Foreign Medical Graduates. In 2015, US citizens made up 26% of all IMGs seeking certification in the US [2]. A critical step for certification is passing the US Medical Licensing Examinations (USMLEs). A 2006 report demonstrated that US medical graduates (US MGs) received higher scores on the USMLE steps 1 and 2 than IMGs and that US IMGs scored below US MGs and non-US IMGs [3]. One exception was the USMLE clinical skills (CS) examination, on which US IMGs had a higher pass rate than non-US IMGs [2]. Regardless, the USMLE step 2 CS remains a high-stakes endeavor for US IMGs, with an overall first-time pass rate of 80%, compared to 96% for US MGs [4]. Taking the exam is associated with considerable cost and anxiety. Although the value of the USMLE step 2 CS exam to residency programs and learners has been questioned [5], its value to the public of ensuring that individuals seeking to train and practice in the US meet minimum standards of competence in communication and physical examination skills, clinical reasoning, and spoken English proficiency may have more importance when applied to IMGs.

US medical schools have adjusted CS training in response to the USMLE step 2 CS examination, including increased use of standardized patients (SPs) and simulations [6]. US and Canadian students matriculating at foreign medical schools may be at a disadvantage for the USMLE step 2 CS examination due to less exposure and training with SPs and objective structured clinical exam experiences; a decreased emphasis on structured patient-centered communication skills training, in particular around complex skills such as shared decision-making, delivering bad news, and handling emotions; fewer opportunities to be directly observed in the clinical setting [3]; and less role modeling of patient-centered communication skills.

Multiple USMLE step 2 CS preparation courses exist in the US, but these courses are expensive, require students to travel from their existing learning environment, and may be more focused on ‘teaching to the exam’ rather than developing competence in patient-centered communication skills. We describe the development and evaluation of a USMLE step 2 CS preparation course provided at the Technion Israel Institute of Technology School of Medicine for US and Canadian medical students in the Technion American Medical School (TEAMS) program between 2012 and 2016.

## Methods

### Course development

The goals of the course were to improve participants’ (1) knowledge of the format and content of the USMLE step 2 CS examination and comfort with SP encounters; (2) patient-centered communication skills; (3) ability to perform a focused history with a SP; and (4) ability to complete a USMLE step 2 CS examination post-encounter note. The 2-day course employed didactic and experiential learning methods to promote deliberate practice. Patient-centered communication instruction was modeled on the elements of communication for which there is broad consensus [7]. Other course components were based on published information on the exam, evidence-based medical education, and our own extensive experience teaching CS in the US [8,9]. On day 1, students received an overview of the exam format and strategies for patient-centered interviewing, performing a focused physical exam, and completing the post-encounter note. Students engaged in role play and received peer and faculty feedback using a structured observation guide. On day 2, students completed 3 video-recorded timed mock exam stations in which they performed a focused history and completed a post-encounter note. Fourth-year TEAMS medical students served as SPs. Cases were developed by the course faculty based on likely exam scenarios. Following each encounter, students received structured feedback from the SP on their interpersonal and communication skills. Students then reviewed 2 of the 3 videos in two 35-minute, one-onone sessions with course faculty (RBL, DC). During these sessions, students used a structured template to guide self-assessment, record feedback, and develop an individualized learning plan for independent examination preparation.

### Setting and course participants

The course participants were third-year students at TEAMS, an international 4-year medical program located at the Technion Medical School in Haifa, Israel. The 4-year curriculum is taught in English. The program accepts applicants who are US and Canadian citizens or permanent residents who have spent at least 8 years out of the last 10 years residing in North America. TEAMS students have all completed a 4-year college premedical curriculum in the US or Canada. The program offers an opportunity for students to pursue an MD (doctor of medicine) educational program with a curriculum and course of study patterned after US medical schools. The application requirements are similar to those of North American medical schools and include MCAT (Medical College Admission Test) scores, academic transcripts, and letters of recommendation. The average class size is 30 students per year. The preclinical curriculum consists of basic science courses and an introduction to CS. The final 2 years consist of clinical rotations in both Israel and the US. Participation in the course was voluntary, and students paid course tuition.

### Course evaluation

We hypothesized that students’ confidence and competence would increase and that the overall pass rate for the TEAMS program would improve. Students were asked to complete pre- and post-course online surveys addressing their knowledge of the USMLE step 2 CS examination format, comfort with performing a timed SP encounter, and overall confidence and competence with the CS tested. We collected demographic information including age, gender, and country of birth. The survey included a Likert scale and open-ended responses. Emails inviting students to complete the surveys stated that responses would be confidential and asked students to select an ‘opt out’ response if they did not want their responses used for research. An administrative assistant with no other role in the course or TEAMS program had access to de-identified data. Students were contacted by email up to 3 times in order to increase the response rate. The course evaluation plan and surveys were reviewed through the Technion Internal Review Board and deemed exempt from further review after informed consent was received from the participants.

### Statistical analysis

All items measuring overall knowledge and confidence in taking and passing the USMLE step 2 CS examination, comfort with the SP encounter, and confidence and competence in specific CS were measured on a 5-point Likert scale (0, poor; 1, below average; 2, average; 3, above average; 4, outstanding). A mean score was computed for each student at pre- and post-course time points. All single-item and summary score measures fell within acceptable limits of skew and kurtosis.

To test whether students’ self-reported measures of knowledge, preparation, confidence, and competence in CS increased between the pre- and post-course responses, we computed the paired t-test for each of the 22 single-item measures. To test whether pre- to post-course changes in confidence and competence in skills varied according to gender or country of birth, we used repeated-measures analysis of variance (ANOVA) with summed confidence and competence scores, respectively, as the dependent variable. Time (pre versus post) by gender and country of birth interaction terms tested hypotheses related to demographic differences. For country of birth, a categorical variable was created to compare the total scores for students born in the US, Canada, and other countries. We compared the overall pass rates provided by the TEAMS program with data publicly available on the USMLE website [4].

We began evaluation of the course in 2013, using our 2012 course as a pilot. We completed an analysis of survey items for course participants during the years 2013–2016. We did include the pass rate from 2012. All analyses were performed in IBM SPSS ver. 24.0 (IBM Corp., Armonk, NY, USA).

## Results

Ninety students have participated since 2012, with 76 participating during the evaluation period between 2013 and 2016. There were no significant demographic differences between participants and nonparticipants. The raw data are available in Supplement 1. Of the 76 participants, 73 gave their permission to use their course evaluation for research. Nine of the consenting students did not complete the post-course survey. Table 1 presents the demographic characteristics of the remaining sample of 64 students who completed pre- and post-course surveys. A slight majority were male, and most were US-born.

Among the 64 respondents who completed pre- and post-course surveys, most items were missing a negligible number of responses (0 to 1). All available data for each pair of pre-post measures were included. The results of paired t-tests comparing pre- to post-course changes on USMLE self-ratings are compiled for each item in Table 2.

Students reported significant increases in confidence and competence scores across all clinical skill items and averaged scores (Table 3). Repeated-measures ANOVA indicated that the increase in mean CS confidence scores did not differ according to gender (interaction of gender× time F(1, 62)= 0.042, P= 0.84), or country of birth (interaction of country of birth× time F(2,59)= 1.05, P= 0.36). The mean competence scores did not vary by gender (interaction of gender× time F(1,57)= 1.08, P= 0.30) or country of birth (interaction of country of birth× time F(2,54)= 0.26, P= 0.77).

**Fig. 1.**
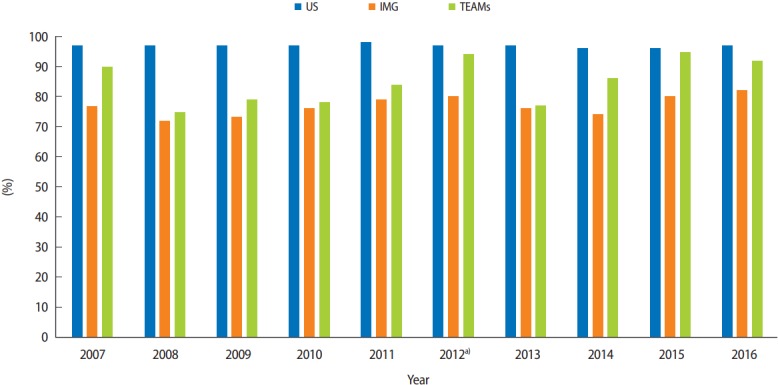
USMLE step 2 CS exam pass rates for US, IMG, and TEAMS students, 2007–2016. USMLE, United States Medical Licensing Exam; CS, clinical skills; US, United States; IMG, international medical graduate; TEAMS, Technion American Medical School. ^a)^USMLE course begins.

The average TEAMS USMLE step 2 CS pass rate from 2007 to 2011 was 82%, while the average pass rate of the 2012–2016 course participants was 89%. The pass rate for students in the course was 95% and 92% in 2015 and 2016, respectively. Fig. 1 presents the USMLE step 2 CS pass rates for US graduates, IMGs, and TEAMS students from 2007 to 2016.

**Fig. 2.**
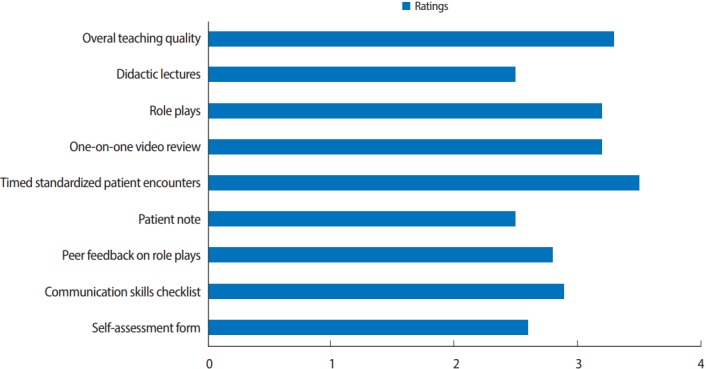
Learner ratings of the usefulness of educational methods used in the course. Mean scores on a 5-point Likert scale (0, poor; 1, below average; 2, average; 3, above average; 4, outstanding).

The ratings of teaching methods were compiled, and mean scores for the overall ratings of teaching quality and specific teaching methods are presented in Fig. 2. Interactive, experiential teaching methods received the highest ratings.

## Discussion

This is the first study to describe a successful USMLE step 2 CS preparation course conducted within the local learning environment of an international medical school. This course significantly improved students’ confidence and perceived competence in their ability to pass and perform specific CS related to the exam. Over 5 years, there was a trend towards overall improved pass rates for TEAMS students; the pass rate approached that of US MGs and was higher than IMGs overall. There is currently a dearth of published descriptions or evidence regarding commercial and noncommercial USMLE step 2 CS preparation courses. A limited number of published studies evaluating the impact of commercial preparation courses have focused on the USMLE step 1 exam; these studies are methodologically limited, and have demonstrated little to no impact on test scores [10].

IMGs continue to make up a significant proportion of practicing US physicians and should have access to CS training that not only prepares them for the USMLE exams, but also for the care of patients in the US. Additionally, with the availability of the Accreditation Council for Graduate Medical Education’s international accreditation program, an opportunity has emerged for strengthening CS training across the international medical education continuum [11]. An individual’s performance on the data interpretation and communication and interpersonal skills sections of the USMLE step 2 CS examination is positively correlated with ratings of history taking and physical examination during internship [9]. Ideally, CS training in preparation for the USMLE step 2 examination would also improve long-term practice and meaningful health outcomes.

Another important outcome of this program that may have helped to improve pass rates is improved student confidence and comfort, which may decrease test anxiety. Test-taking anxiety is modestly inversely correlated with USMLE step 1 performance and can be reduced [12]. We incorporated methods shown to reduce stress and potentially improve performance, such as mental rehearsal [13]. As students in the TEAMS program do not have the opportunity to participate in many formative or summative SP encounters, the ability to increase familiarity with that format may increase their comfort and reduce their cognitive load during the exam. In addition, using peers as SPs may reduce stress and improve learning [14].

Conducting this course at the local institution with input from students and program directors allowed us to better understand students’ local educational environment and tailor our teaching to students’ needs. A positive, supportive environment that invites learners to share their strengths and areas for improvement promotes learning. Similarly, a learning community in which students are intentionally engaged in learning from each other activates the social aspects of learning and encourages students to challenge themselves and take risks with their learning. TEAMS classes are typically small and students spend a significant amount of time together. We leveraged this asset and emphasized a team learning approach while promoting a supportive learning environment.

This study has limitations. First, this was a single-institution study that has not been replicated elsewhere. Second, while this course cost less to participate in than commercially available courses, the cost was still significant, and this factor may limit the generalizability of our findings. However, the course allowed students to remain within their existing learning environment where they may have benefited from collaborative learning with peers, as discussed above. Third, this course primarily focused on teaching communication skills, but the physical exam and the patient note tended to be low-scoring components for many examinees [15]. Lastly, while we were able to demonstrate an overall increase in the pass rate, we cannot prove causality. There may have been other factors, both internal and external, that impacted pass rates. In 2013, TEAMS was undergoing a curricular change, which might explain the low pass rate for that year. Similarly, the USMLE step 2 CS grading scale has changed over time, potentially confounding our comparison of pass rates.

This study is the first to describe a USMLE step 2 CS preparation course specifically designed for US IMGs that was delivered within the students’ local learning environment. The use of experiential learning and instruction focusing on patient-centered communication skills improved students’ confidence and perceived competence, and a trend toward improved pass rates on the USMLE step 2 CS examination was observed. This type of course may help to close the gap between US MG and IMG pass rates on a high-stakes licensing exam and potentially promote the long-term retention of CS.

## Figures and Tables

**Table 1.** Demographic characteristics of the 64 students who participated in the United States Medical Licensing Exam step 2 clinical skills exam preparation course and provided pre- and post-survey data

Characteristic	Value
Gender (male)	34 (53)
Age (yr)	26 ± 2 (21–33)
Country of birth	
United States	37 (58)
Canada	15 (23)
Other	10 (16)
Missing	2 (3)
Year course completed	
2013	19 (30)
2014	8 (13)
2015	15 (23)
2016	22 (34)

Values are presented as number (%) or mean±standard deviation (range).

**Table 2.** Paired t-tests of pre- to post-course changes in mean scores for knowledge and perceptions of the USMLE step 2 CS exam (N=64)

Survey item	Pre-course	Post-course	P-value
Overall knowledge of the structure and format of the USMLE step 2 CS exam.	1.98 ± 0.93	3.20 ± 0.51	< 0.001
How well prepared do you feel to take the USMLE step 2?	0.88 ± 0.49	2.28 ± 0.75	< 0.001
Overall confidence with your ability to pass the USMLE step 2.	1.70 ± 0.81	2.73 ± 0.60	< 0.001
Comfort with performing a timed standardized patient encounter.	1.63 ± 0.79	2.56 ± 0.61	< 0.001

Values are presented as mean±standard deviation. Items were rated using a 5-point Likert scale (0, poor; 1, below average; 2, average; 3, above average; 4, outstanding). USMLE, United States Medical Licensing Exam; CS, clinical skills.

**Table 3.** Paired t-tests of pre- to post-course changes in self-ratings of confidence and competence in clinical skills domains tested on the USMLE step 2 CS exam (N=64)^a)^

Survey item	N	Pre-course	Post-course	P-value
Taking a focused history				
Confidence	64	2.08 ± 0.72	2.72 ± 0.60	< 0.001
Competence	59	2.12 ± 0.70	2.63 ± 0.61	< 0.001
Opening the interview				
Confidence	64	2.45 ± 0.91	3.33 ± 0.59	< 0.001
Competence	59	2.49 ± 0.84	3.27 ± 0.61	< 0.001
Gathering patient data				
Confidence	64	2.33 ± 0.71	2.83 ± 0.55	< 0.001
Competence	58	2.29 ± 0.70	2.86 ± 0.58	< 0.001
Building the relationship				
Confidence	63	2.62 ± 0.73	3.21 ± 0.54	< 0.001
Competence	59	2.53 ± 0.77	3.19 ± 0.57	< 0.001
Sharing information with the patient				
Confidence	64	2.09 ± 0.89	2.84 ± 0.62	< 0.001
Competence	58	2.07 ± 0.79	2.88 ± 0.65	< 0.001
Reaching agreement on problems and plans				
Confidence	64	2.08 ± 0.84	2.94 ± 0.59	< 0.001
Competence	59	2.05 ± 0.80	2.90 ± 0.61	< 0.001
Providing closure to the interview				
Confidence	64	2.00 ± 0.89	2.80 ± 0.72	< 0.001
Competence	58	1.93 ± 0.90	2.79 ± 0.72	< 0.001
Performing a focused physical examination				
Confidence	64	1.80 ± 0.69	2.11 ± 0.78	0.009
Competence	59	1.80 ± 0.66	2.12 ± 0.67	0.002
Completing a patient note				
Confidence	64	1.19 ± 0.83	2.08 ± 0.84	< 0.001
Competence	59	1.31 ± 0.86	2.02 ± 0.90	< 0.001
Mean score across all items				
Confidence	64	2.07 ± 0.54	2.76 ± 0.42	< 0.001
Competence	59	2.07 ± 0.54	2.79 ± 0.42	< 0.001

Values are presented as mean±standard deviation. Items were rated using a 5-point Likert scale (0, poor; 1, below average; 2, average; 3, above average; 4, outstanding). USMLE, United States Medical Licensing Exam; CS, clinical skills.

a) Minimal missing responses for competence ratings.

## Supplementary materials

Supplement 1. Data file is available from https://doi.org/10.7910/DVN/XUFWM8.

Supplement 2. Audio recording of the abstract.
